# Discrepancies in Open Access Fees within Pharmacology, Toxicology, and Pharmaceutics Journals

**DOI:** 10.34172/apb.2023.076

**Published:** 2023-04-29

**Authors:** Rana M.F. Sammour, Aliasgar Shahiwala

**Affiliations:** Pharmaceutics Department, Dubai Pharmacy College for Girls, Dubai, UAE.

**Keywords:** Open access, Q ranking, Impact factor, Publication fees

## Abstract

Modern science has been transformed by open access (OA) publishing levied a significant economic burden on the authors. This article analyzes the discrepancies among OA publication fees in pharmacology, toxicology, and pharmaceutics. The observations comprise 160 OA journals and their corresponding Q ranking, SJR, H index, impact factor, country, and cost of publication. The OA fees were found to depend on the quality matrices, which was unexpected. Differences in OA fees raise ethical questions as OA fees are meant to cover the publication charges by the publishers or generate more revenues by taking advantage of the authors’ temptation to publish in high-impact journals. Despite our findings being based on limited sample size and belonging to a particular field (pharmacy), it will shed considerable light on the issue of discrepancies among APCs charged by OA journals.

## Introduction

 The conventional approach of scientific publishing, which entails charging readers subscription fees or usage fees to access scientific content, has been under intense criticism for a long time.^1^ Key concerns are that publishers control access to the findings of taxpayer-funded research and use them for their financial gain.^2^ Although scientists offer their work for free, readers must pay to read it.

 With the emergence of digital tools and publishing technologies, the scholarly communications sector has transformed from traditional journals to open access (OA) journals. The OA model grants free access to the readers compared to the traditional subscription model, which requires the readers to pay to access scholarly information. OA increases the visibility and reuse of academic research necessary to advance science without financial, legal, or technical barriers to accessing scientific knowledge. The Public Library of Science (PLoS) and BioMed Central (BMC) started the OA model by charging article processing charges (APC) to the authors, their institutions, or funders to cover the cost of publishing in 2000.^3^ Since then, many leading traditional and new publishers have started applying the OA model.

 There could be many factors for the increased presence of OA journals. Still, the tremendous increase in such journals is mainly due to the “Publish or Perish” dynamic in academics.

 Scientific and scholarly communities have growing awareness and concern about the business models and operating practices adopted by publishers, mainly commercial publishers. The OA model necessitates authors to pay for publishing, unlike the subscription model, which demands readers pay for the content they read. This creates a barrier for researchers to publish their work without access to sufficient funds. Also, APC journals may compromise their quality standards to publish as many articles as possible if they are forced to charge writers for publishing their work. Moreover, expanding predatory journals is another threat to researchers and scientists. Predatory journals are periodicals that use the open-access publishing hypothesis to profit at the cost of excellence and academic integrity. These journals frequently publish articles without concern for scientific accuracy or relevance, without thorough peer review, and with minimal or no editorial standards. They provide inaccurate or misleading information, depart from best editorial and publication practices, and lack transparency.^4^

 Usually, predatory journals are indexed less frequently than traditional journals. It can be challenging to find research published in them through database searches. This suggests that when good research is published in predatory journals, patient participation, animal use, and funding for the study, including paying APCs, may be wasted.^5^

 Predatory publication must be combated by ongoing, variable efforts. As long as institutions utilize a scholar’s output of papers as a criterion for graduation or career promotion, the risk is unlikely to go away. Predatory publications thrive in environments that encourage the “publish or perish” mentality, lack of awareness of predatory publishing, and difficulty distinguishing between legitimate and illegitimate journals.^6^

 To limit this threat’s spread, all papers intended for publications should undergo systematic review, including those published in predatory journals. Moreover, systematic reviews should use standardized practices. They should also conduct rigorous risk of bias analyses to ascertain the articles that may affect the scientific findings.^5^

 There is no doubt that OA literature is not free to produce; however, the cost of production of OA is less than subscription journals as the cost associated with the printing and setting up of subscription paywalls is not there.^7^ Scientists whose sponsors or universities won’t or can’t cover the costs are often discouraged from publishing in journals because of article processing charges (APCs), per-paper author fees for journals supporting OA publishing. The Directory of Open Access Journals (DOAJ) has listed 18300 OA journals, of which 5830 journals (31.86%) charge APCs.

 However, a significant variation is observed in the OA fees or APCs among various journals and disciplines. In 2010, APCs ranged from $8 to $3900, the highest by high-impact journals from major international publishers and the lowest by journals published in developing countries.^3^ APCs are the highest in medicine (47%) and the lowest in arts (0%).^8^ Also, usually, high-prestige journals charge more fees.^9^ This will prevent the authors without the funds from dreaming of such journals.

 This manuscript examines discrepancies in the journals’ APCs based on criteria such as journal quartile, country of publication, h-index, and SJR. Since evaluating all the journals (over twenty thousand) is nearly impossible, we have limited our inclusion criteria to only journals related to the pharmacy field.

## Methods

###  Data Collection

 This study focuses on the pharmacy field in which there has been a proliferation of OA journals. The observations comprise 160 pharmacy-related OA journals indexed in the SCImago Journal and belong to the areas of pharmacology, toxicology, and pharmaceutics. The data were collected during April-May 2022. The Journal name and its corresponding Q ranking, SCImago Journal Rank (SJR) indicator, H index, impact factor, and country of publication were compiled. Then, the open-access fee was found by referring to the journal website. To analyze the data using parametric tests, the sample size was kept to forty in each Q category, which is well above the standard norm of thirty. IBM SPSS Statistics for Windows, version 26 (IBM Corp., Armonk, NY, USA) was used for statistical data analysis.

## Results and Discussion

 Different metrics have been developed to evaluate scholarly journals’ impact based on citations. The SJR is a size-independent web-based metric designed to assess the current “average prestige per paper” of journals.^10^ According to Scopus^®^ Classification, journals are classified into 309 distinct subject groups and twenty-seven key thematic categories. The data on the top forty SJR journals from each quartile were analyzed. The sample size of forty was selected as it is possible to use parametric procedures even when data are not normally distributed.^11^

 A quartile in Scopus refers to a group of reputable scientific journals. The quartile displays how popular the journal is among scientists according to the least and most cited journals. They are categorized into quartiles, with Q1 having the highest credibility and Q4 having the lowest.

**Figure 1:**
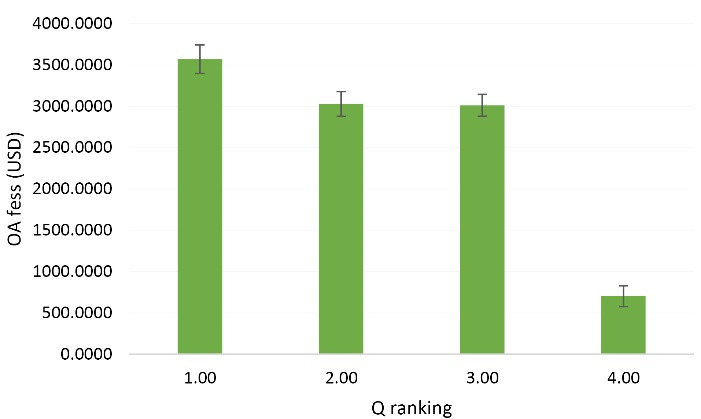
OA fees according to Q ranking (mean ± SD)

Figure 1 compares the APC difference based on Q ranking. As evident, the pricing becomes significantly lower as the Q ranking decreases further from Q1. The APC range, according to Q-ranking, was 6700-950, 4750-950, 4190-100, and 3390-0 for Q1, Q2, Q3, and Q4, respectively. Between the APCs of Q2 and Q3 journals, no significant difference was observed.

**Figure 2:**
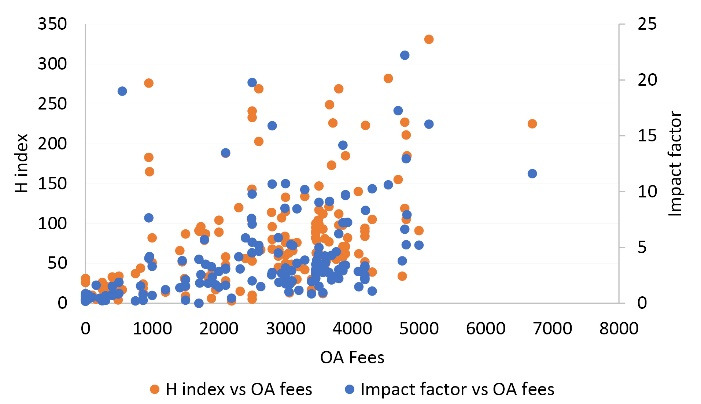
Comparison of OA fees against H index and impact factor

Figure 2 suggests a highly significant correlation (*P* < 0.01) was found for the H index and impact factor against OA fees. The average APCs are in descending order from high to low impact in the SCOPUS and nonindexed journals.^3^ With the increase in the H index and impact factor, publishers charge high OA fees.

 Twenty-four countries participated in publishing the first forty journals in each Q category (Table 1). UK published most journals in the Q1 category, followed by the US and the Netherlands. The remaining countries have either no publications or only one publication (4 countries) in the Q1 categories.

**Table 1:** Country-wise listing of Journals in different quartiles (n = 160)

**Country**	**Q ranking**				**Total**
	**1.00**	**2.00**	**3.00**	**4.00**	
Argentina	0	0	0	1	1
Australia	1	0	0	0	1
Bangladesh	0	0	0	1	1
Brazil	0	0	0	1	1
Canada	0	0	1	0	1
China	0	0	0	1	1
France	0	0	1	1	2
Germany	0	1	0	2	3
India	0	0	0	6	6
Iran	0	0	0	4	4
Iraq	0	0	0	1	1
Ireland	1	1	0	0	2
Italy	0	0	1	0	1
Japan	0	2	0	0	2
Netherlands	5	3	3	4	15
New Zealand	0	0	1	0	1
Nigeria	0	0	0	2	2
Poland	0	0	0	1	1
South Korea	0	0	0	4	4
Switzerland	1	3	2	0	6
Thailand	0	0	0	2	2
United Arab Emirates	1	4	3	7	15
United Kingdom	18	11	12	0	41
United States	13	15	16	2	46

 OA fees according to the country of publications are shown in Table 2. Only countries with a stake in all Q categories are included; hence, data are limited to only developed countries. Significant differences were found in OA among the countries except for the US and the UK, and OA fees from the Netherlands are found to be highest in all four categories among the four countries compared. Within the country of publication, no significant (*P* > 0.05) were found among the Q1, Q2, and Q3 categories. The significant difference (*P* < 0.05) in APCs was found only between Q4 and the first three categories. Surprisingly, average OA fees for Q3 were higher than Q2 journals published by the UK, although the difference was not significant between the categories.

**Table 2:** OA Fees according to the q-rank and country of publication

**Country**	**Q ranking**			
	**1.00**	**2.00**	**3.00**	**4.00**
USA	3442.69 ± 1164.62	3200.67 ± 896.90	2893.81 ± 932.70	500 ± 353.55
UK	3520 ± 1186.54	2893.09 ± 957.85	3425.83 ± 665.04	NA
UAE	3930*	2625 ± 1151.19	3455 ± 0	893.57 ± 431.13
Netherlands	4306 ± 732.41	3476.67 ± 390.17	2948.33 ± 908.05	1263.75 ± 873.70

*Only one journal listed.

 Economic theory suggests that the price of a commodity should be the same across the world if other factors, such as transaction costs, transportation costs, government taxes, legal restrictions, currency exchange rates, and inflation rates, which is hardly true in the real world. The data shows that publishers in developed countries dominate the OA journals. The funding levels for scientists in developing countries are usually lower,^12^ which deprives them of publishing in high-impact journals due to their high APCs.^13^ However, there are a few exceptions where some publishers offer waivers on APCs for scientists of developing and poorly developed countries.^14^

 OA fees have continuously risen, making publishing difficult for scientists with fewer financial resources. The mean APC for 739 APC-funded publications increased by 50% between 2010 and 2019. OA journals with more frequently cited publications have higher article processing fees.^15^ Similar to any business model, the companies of large subscription journal publishers integrate OA journal publishers that have already achieved significant success in their companies.^15^ As a result, the DOAJ’s nine largest publishers saw their APC revenues rise by more than half between 2019 and 2020.^16^

## Conclusion

 OA has significant potential for expediting the recognition and dissemination of research findings. Although OA journals are less expensive than traditionally published literature, authors know they are not free to generate. However, the differences in OA fees also raise ethical questions as OA fees are meant to cover the publication charges by the publishers or generate more revenues by taking advantage of the authors’ temptation to publish in high-impact journals. Despite our findings being based on limited sample size and belonging to a particular field (pharmacy), it will shed considerable light on the issue of discrepancies among APCs charged by OA journals. Based on the inconsistencies seen among the OA fees of the journals belonging to the same ranking, authors can evaluate the service they are getting for the fee they are paying.

## Competing Interests

 There is no conflict of interest.

## Ethical Approval

 Not applicable.
